# Live fish in the trachea and bronchus: a case report

**DOI:** 10.1186/1756-0500-7-658

**Published:** 2014-09-18

**Authors:** Kanu Lal Saha, Debesh Chandra Talukder, Mohammad Asaduzzaman Rasel, Anamika Saha

**Affiliations:** Department of Otolaryngology and Head-Neck Surgery, Bangabandhu Sheikh Mujib Medical University, Dhaka, Bangladesh; Department of Otolaryngology and Head-Neck Surgery, Dhaka Medical College Hospital, Dhaka, Bangladesh; Department of Paediatrics, Dhaka Medical College Hospital, Dhaka, Bangladesh

**Keywords:** Foreign body, Trachea, Bronchus, Live fish, Flat and slippery

## Abstract

**Background:**

A live foreign body in trachea or bronchus is a very rare as well as life threatening condition. A bigger fish with irregular shape usually impacts at the oropharynx, hypopharynx or inlet of the larynx. But a small or flat, elongated foreign body may cross the glottis and enter into trachea and bronchus. We report a rare type of very long live fish in trachea and bronchus.

**Case presentation:**

A 16-year-old Bangladeshi boy presented with severe respiratory distress and cyanosis with a history of live fish impaction in the throat. To relieve respiratory distress and secure life an emergency tracheostomy was carried out while a tail fin of a fish was seen through tracheostome directed to the right bronchus which was grasped with an artery forceps and pulled out of the trachea. Respiratory distress was relieved immediately. The fish removed from the trachea was locally known as Guchi Baim Fish (*Macrognathus pancalus*). It was about 16 cm long and about 2 cm wide at its central region.

**Conclusion:**

Live fish lodging in the trachea and bronchus is an acute emergency condition. It is very difficult to diagnose and manage because of its presence in critical anatomical location. So a quick short history from accompanying persons especially about the type of fish is crucial to predicting the site of its lodgement in the airway as well as management plan. Avoidance of the tendency of holding the fish between teeth during fishing can prevent this life threatening condition.

## Background

A live foreign body in trachea or bronchus is a very rare as well as life threatening condition. Usually a foreign body reaching the inlet of larynx is coughed out due to highly sensitive and protective cough reflex. But a small or flat, elongated foreign body may cross the glottis and enter into trachea and bronchus. Among reported cases of whole live fish impacted in the air passages Koi fish [1–3] is common, particularly in the South Asia region. Two Reef –fish [4] and a Talapia fish [5] impacted above the inlet of larynx were reported from Papua New Guinea and a Lepomis macrochirus [6] in the hypopharynx was reported from the USA. A Todi fish [7] impacted in the trachea and bronchus was reported from India. We are reporting a case of Guchi Baim fish in trachea in a young boy. Till date this is the first reported case in Bangladesh and reportedly the longest live fish in the trachea.

## Case presentation

**Figure 1:**
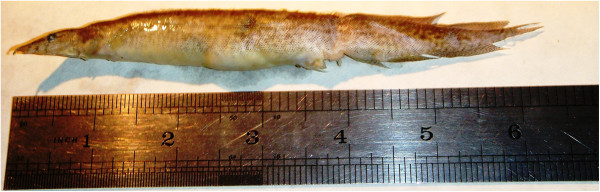
**Guchi Baim fish removed from trachea and bronchus.**

A 16-year-old Bangladeshi boy was brought to the Department of Otolaryngology of Dhaka Medical College Hospital in March 2010 with severe respiratory distress and cyanosis. A short history from his father suggested he might have impacted a live fish in his throat. A quick examination of the throat was carried out and some lacerated areas were found in the oral cavity, oropharynx and hypopharynx. No foreign body was found but the patient was in severe distress, vigorously coughing and unable to talk. To relieve respiratory distress and secure life, an emergency tracheostomy was carried out under local anaesthesia. Still, respiratory distress was not relieved. A quick examination of the inside of lower trachea through the tracheostome revealed a tail fin of a fish directed to the right side of the bronchus. It was grasped with an artery forceps and pulled out of the trachea. Respiratory distress was relieved immediately. The patient was moved to the recovery room and monitored. The fish removed from the trachea was locally known as Guchi Baim fish (*Macrognathus pancalus*) (Figure 1). It was about 16 cm long and about 2 cm wide at its central region. It was very slippery.

After the emergency procedure, detailed history was taken from the parents. They said that on the Eid day (a Muslim religious festival), the boy with his friend had gone to a nearby canal to catch fish. He found two fishes at a time, and at one point held one’s head between his teeth while trying to catch the other one, keeping both hands free. Then suddenly the live moving fish slipped into his mouth and the boy immediately rushed home. Initially his family members saw the fish in his mouth and tried to pull it out with hands by holding the tail end, but couldn’t do so, as it was live, moving and very slippery. The fish slipped downwards beyond their vision. Then they rushed to the nearby Upazilla Health Complex from where the boy was referred to a District Hospital. At the District Hospital, attending doctors failed to remove it and as respiratory distress kept increasing, they immediately referred him to Dhaka Medical College Hospital (a tertiary level medical facility). Already five valuable hours had passed from the onset of incident to removal of the fish at the hospital. Probably he is one of the lucky survivors of this kind of incidence. Tracheostomy was closed within seven days and the patient was discharged from hospital.

The case report was approved by the ethical review committee of Dhaka Medical College.

## Discussion

In many countries fishermen often kill fish by crushing the head between teeth or hold the fish between their teeth to keep both hands free to catch the next one, while gathering fishes from the net. But sometimes struggling fish slips into mouth and lodges in the pharynx, oesophagus, larynx or tracheobronchial tree. Final destination of a live foreign body depends on the size, shape and initial presentation of impaction in the airway. Sign and symptoms of swallowed foreign body vary depending on the size and shape. Large, irregular shape fish occupying in the oropharynx, hypopharynx produces less initial severe respiratory symptoms. Flat and smaller foreign body may lodge in the upper trachea and bronchus causes increase in respiratory distress in course of time. But large foreign body impacted in these areas may cause severe distress and quicker causality. Especially if the live fish initially impacts in the inlet of larynx or trachea death is sometimes inevitable even before transfer the patient to the hospital [8]. Reported cases of Koi fish [1–3], Reef fish [4], Talapia fish [5], Lepomis macrochirus [6] were found in the oropharynx, hypopharynx or inlet of the larynx. In 1973 Tarasia and Mishra [7] reported a case of 10 cm long Todi Fish (Macrognathus saculeatum) in the trachea and left bronchus of a 10 year old boy. The fish was retrieved from the trachea and the boy made an uneventful recovery. No tracheostomy was done. Our case, Guchi Baim Fish (*Macrognathus pancalus*) is similar to above reported case [7] which was flat, elongated, slippery, and easily crossed the glottis and entered trachea and right bronchus. In most reported cases [1–6] part of live fish usually the tail end was seen in oral cavity, oropharynx or laryngopharynx during physical examination. If the foreign body in the back of the throat is visible and approachable it can be removed easily and further complications can be avoided. But attempts of removal of foreign body may be dangerous. Especially if it slips and drops down to inlet of larynx symptoms may aggravate quickly and be fatal. In our case initially the fish was in the oral cavity. During attempts of manual removal the moving fish slipped and dropped down it to trachea and bronchus. In case of live fish in trachea or bronchus physical examination reveals nothing except some laceration in oral cavity and pharynx. On the other hand severe respiratory distress limits the scope for endoscopic examination. Though radiographs are important clinical adjuncts in the identification of swallowed foreign bodies regarding its size and shape especially in radio opaque type, in our case it would give us little information about the shape and size of live fish as it was a soft tissue. Besides that no portable X-ray facilities were available and sending the patient of severe respiratory distress to X-ray room would be a risky decision. So we did not do radiography in our case. Besides this a quick short history from accompanying persons especially about the type of fish is crucial to predicting the site of its lodgement in the airway. Usually emergency tracheostomy is recommended to relieve respiratory distress and to secure life. If respiratory distress is not relieved immediately after tracheostomy, careful quick examination of lower trachea must be done to check presence of any foreign body or its part. Sometimes the only way to find and remove a foreign body is by holding its part.

Civil surgeon (district administrative chief of health system) of the area from where initial management started was informed about the issue so that awareness can be built up among the fishermen to avoid such accidental causality.

## Conclusion

Live fish lodging in the trachea and bronchus is an acute emergency condition. It is very difficult to diagnose and manage because of its presence in critical anatomical location. So a quick short history from accompanying persons especially about the type of fish is crucial to predicting the site of its lodgement in the airway as well as management plan. Avoidance of the tendency of holding the fish between teeth during fishing can prevent this life threatening condition.

## Consent

Written informed consent was obtained from the patient’s parents for publication of this Case Report and any accompanying images. A copy of the written consent is available for review by the Editor-in-Chief of this journal.
